# Clustering of lifestyle risk factors in relation to suicidal thoughts and behaviors in young adolescents: a cross-national study of 45 low- and middle-income countries

**DOI:** 10.1186/s44263-024-00055-4

**Published:** 2024-04-12

**Authors:** Yongle Zhan, Pei Wang, Yongan Zhan, Zhiming Lu, Yidan Guo, Noor Ani Ahmad, Andrew Owusu, Tepirou Chher, Johnson T. Hinneh, Krishna Kumar Aryal, Noorali Darwish, Sameera J. Senanayake, Bushra abdulrahman Ahmed Mufadhal, Alissar Rady, Marcia Bassier-Paltoo, Suvd Batbaatar

**Affiliations:** 1 Department of Surgery, LKS Faculty of Medicine, The University of Hong Kong, Hong Kong, China; 2 Department of Statistics, Miami University, Oxford, OH USA; 3 School of Mathematics, Sun Yat-Sen University, Guangzhou, China; 4 Institute of Basic Medical Sciences, Chinese Academy of Medical Sciences, School of Basic Medicine, Peking Union Medical College, Beijing, China; 5 School of Mathematical and Statistical Sciences, Clemson University, Clemson, SC USA; 6 Institute for Public Health, Ministry of Health, Putrajaya, Malaysia; 7 Department of Health and Human Performance, Middle Tennessee State University, Murfreesboro, TN USA; 8 Preventive Medicine Department, Ministry of Health, Phnom Penh, Cambodia; 9 Ministry of Education, Monrovia, Liberia; 10 Bergen Centre for Ethics and Priority Setting in Health, University of Bergen, Bergen, Norway; 11 Ministry of Public Health of Afghanistan, Kabul, Afghanistan; 12 Australian Centre for Health Services Innovation, Queensland University of Technology, Brisbane, QLD Australia; 13 Consultant of Public Health, School Health & Nutrition, Sana’a, Yemen; 14 World Health Organization Country Office, Beirut, Lebanon; 15 Health Programs and Delivery Division, Ontario Ministry of Health, Toronto, ON Canada; 16 National Center for Public Health, Ministry of Health, Ulaanbaatar, Mongolia

**Keywords:** Lifestyle risk behavior, Suicidal ideation, Suicidal plan, Suicidal attempt, Cross-national, Adolescent, LMIC, Sufficient dimension reduction, Latent class analysis, Risk stratification

## Abstract

**Background:**

Prior research has reaffirmed lifestyle risk behaviors to cluster among adolescents. However, the lifestyle cluster effect on suicidal thoughts and behaviors (STBs) was unclear among adolescents in low- and middle-income countries (LMICs). No comparison of such associations was conducted across nations.

**Methods:**

Data from 45 LMICs were obtained from the Global School-based Student Health Survey (GSHS) between 2009 and 2019. Lifestyle behavior factors were collected through a structured questionnaire. Suicidal ideation, plan, and attempt were ascertained by three single-item questions. Lifestyle risk scores were calculated via a sufficient dimension reduction technique, and lifestyle risk clusters were constructed using a latent class analysis. Generalized linear mixed models with odds ratio (OR) and 95% confidence interval (CI) were used to estimate the lifestyle-STB associations.

**Results:**

A total of 229,041 adolescents were included in the final analysis. The weighted prevalence of suicidal ideation, plan, and attempt was 7.37%, 5.81%, and 4.59%, respectively. Compared with the favorable lifestyle group, the unfavorable group had 1.48-, 1.53-, and 3.11-fold greater odds of suicidal ideation (OR = 1.48, 95%CI: 1.30–1.69), plan (OR = 1.53, 95%CI 1.34–1.75), and attempt (OR = 3.11, 95%CI 2.64–3.65). Four clusters of lifestyle risk behaviors were identified, namely healthy lifestyles (H–L), insufficient intake of vegetables and fruit (V-F), frequent consumption of soft drinks and fast food (D-F), and tobacco smoking and alcohol drinking (S-A) clusters. Compared with H–L cluster, V-F cluster was associated with 43% and 42% higher odds of suicidal ideation and plan, followed by S-A cluster (26% for ideation and 20% for plan), but not significant in D-F cluster (*P* > 0.05). D-F cluster was associated with 2.85-fold increased odds of suicidal attempt, followed by V-F cluster (2.43-fold) and S-A cluster (1.18-fold).

**Conclusions:**

Clustering of lifestyle risk behaviors is informative for risk stratification of STBs in resource-poor settings. Lifestyle-oriented suicide prevention efforts should be initiated among school-attending adolescents in LMICs.

**Supplementary Information:**

The online version contains supplementary material available at 10.1186/s44263-024-00055-4.

## Background

Suicide is a serious public health problem, particularly in adolescents, with a fourth rank of leading cause of death among youth aged 15–19 years old worldwide [1]. As reported by the World Health Organization (WHO), up to 88% of suicide death cases came from low- and middle-income countries (LMICs) [1]. To prevent adolescent suicide, it is imperative to study its immediate precursors, namely suicidal thoughts and behaviors (STBs) including suicidal ideation, plan, and attempt [2]. A prior global population-based study showed that the overall prevalence of the above three STBs reached over 10% among young students aged 13–17 years in LMICs [3]. The high prevalence of adolescent STBs signifies a need for more research focusing on its determinants and interventions.

As adolescence is a critical developmental period for enduring healthy behavior establishment, modifiable lifestyle factors can play a pivotal role in the youth. Hence, the association between adolescent lifestyle factors and STB risk has intrigued researchers in recent years [4]. Soft drink consumption, smoking, alcohol drinking, insufficient physical activity, and sedentary behavior have been examined independently with the risk of STBs [5–9], but their combined influences were far less investigated. Previous studies observed that unfavorable behaviors were likely to co-exist in children. For instance, children who spent excessive time on screen also tended to have poor diet quality [10]. Other examples where unhealthy lifestyles co-occurred included the clustering of engaging less in physical activity and more in sedentary behavior [11]. As risk behaviors do not exist in isolation, examining the clustered effects of these behaviors on adolescent STBs is necessary to aid our understanding and improve our ability to inform screening of suicidality, particularly in LMICs.

At present, the composite score method is one of the widely adopted methods to combine multiple lifestyle risk factors into a summary metric which can be subsequently used as a predictor for health outcomes. For instance, Zhang et al. quantified the number of risk factors into a risk index and found it positively associated with mental problems [12]. However, this index was limited to the equal weighting of all lifestyle factors and did not consider the relationship with the response variable. Latent class analysis is another common method used for behavior clustering. For example, Xiao et al. divided 14,506 U.S. adolescents into four classes and found that those with health-compromising behaviors had a 50% higher risk of suicidal plans compared with those engaging in health-promoting behaviours [13]. Nevertheless, to our knowledge, a similar metric of lifestyle cluster-STB relationship has not yet been reported among adolescents in LMICs.

Therefore, in the present study, we used cross-national data including 45 LMICs from the Global School-based Student Health Survey (GSHS) to evaluate whether STB risks could be stratified by different clusters of lifestyle factors using sufficient dimension reduction technique and latent class analysis. The aims of our study were (1) to determine the country- and individual-level correlates of clustered lifestyle risk behaviors; (2) to estimate the associations between clustered lifestyle risk factors and STB among young adolescents; and (3) to explore the discrepancy of the associations across regions, countries, and population groups in LMICs.

## Methods

### Data sources

Data were obtained and pooled from the GSHS project, which was developed by the World Health Organization (WHO) in collaboration with the United States Centers for Disease Control and Prevention [14, 15] (https://www.who.int/teams/noncommunicable-diseases/surveillance/systems-tools/global-school-based-student-health-survey). GSHS is an ongoing cross-national surveillance survey administered to young on-school adolescents, whose detailed methodology and procedure has been described previously [16]. In brief, the survey consisting of several modules of questions about students’ health behaviors and living circumstances, has been implemented in over 100 countries under a two-stage random cluster sampling design with the same procedure. Three phases (phase one: 2003–2008; phase two: 2009–2012; phase three: 2013–2019) of the GSHS have been deployed. For countries participating in two or more phases, only the latest available one will be included in the present study. To maximize temporal comparability, we restricted datasets whose surveys were conducted between 2009 and 2019, resulting in 57 eligible countries. Countries missing any lifestyle variables were further excluded, remaining 45 countries in the final dataset. Characteristics comparison between included (*n* = 45) and excluded countries (*n* = 12) were performed and no significant difference was observed across country-level and population-level characteristics (*P* > 0.05) (Additional file 1: Table S1).

### Ascertainment of suicidal thoughts and behaviors

STBs for the present study were evaluated by the following three single-item questions: “During the past 12 months, did you ever seriously consider attempting suicide?”; “During the past 12 months, did you make a plan about how you would attempt suicide?”; “During the past 12 months, how many times did you actually attempt suicide?”. According to the STB classification algorithm proposed by Nock [17], participants who answered “yes” to the first question but “no” to the other two questions were considered suicidal ideators; those who answered “yes” to the first two questions but “no” to the third question were considered suicidal planners; and those who ever attempted suicide were considered suicidal attempters (Additional file 1: Fig. S1).

### Assessment of lifestyle risk factors

Eight types of modifiable lifestyle factors, which have been shown to be associated with adolescent STB, were used for analyses, including dietary habits [5, 18, 19], tobacco smoking [6], alcohol consumption [7], physical activity [8], and sedentary behavior [9]. Consumptions of *fruit*, *vegetables*, and *soft drinks* during the past month were categorized as 0, < 1, 1, 2, 3, 4, and ≥ 5 times/day. *Fast food* intake during the past week was categorized as 0, 1, 2, 3, 4, 5, 6, and 7 days. *Tobacco smoking* was dichotomized into never vs. ever. Responses for *alcohol drinking* during the past month were categorized as 0, 1–2 days, 3–5 days, 6–9 days, 10–19 day, 20–29 days, and daily. *Physical active* for ≥ 60 min/day was categorized as 0, 1, 2, 3, 4, 5, 6, 7 days. *Sedentary time* during a typical day was categorized as < 1, 1–2, 3–4, 5–6, 7–8, ≥ 8 h/day.

Each lifestyle risk factor was additionally dichotomized in accordance with international recommendations, WHO guidelines, or previous studies [20–24]: fruit consumption (daily vs. not daily), vegetable consumption (daily vs. not daily), soft drink consumption (not daily vs. daily), fast food consumption (≤ 1 day/wk vs. > 1 day/week), tobacco smoking (< 1 day/month vs. ≥ 1 day/month), alcohol drinking (< 1 day/month vs. ≥ 1 day/month), physical activity (daily vs. not daily), and sedentary time (≤ 4 h/day vs. > 4 h/day).

### Covariates

#### Country-level indicators

We used country income status and geographical area at the survey year (referring to World Bank classifications) [25], the Gross Domestic Product (GDP) per capita, the Human Development Index (HDI), Inequality-adjusted HDI (IHDI), Gender Development Index (GDI), Gender Inequality Index (GII), Current Health Expenditure (CHE) per capita, density of nurses and midwives, and national age-standardized suicide rate at the survey year [26]. In addition, legislation on tobacco and alcohol use, and country’s major religion were used as cultural and contextual indicators.

#### Individual-level indicators

We used age, sex, grade, BMI, proxy of socioeconomic status (SES), loneliness, and sleep problem. The proxy of SES was assessed based on the question “During the past 30 days, how often did you go hungry because there was not enough food in your home?” [27], and was categorized as never, rarely, sometimes, most of the time, and always, representing highest, high, medium, low, and lowest SES status.

### Statistical analyses

All eligible country data were collated into a unified dataset, and all estimates were weighted using the survey’s strata, weights, and primary sampling units (PSUs) to allow the samples nationally representative. The percentages of missing values of all variables of interest were below 20% in the aggregated dataset (Additional file 1: Table S2), and the percentages of missing values of most lifestyle variables were below 20% across countries (Additional file 1: Table S3). The pattern of missingness was assumed to be missing at random (MAR) in the dataset (Additional file 1: Tables S4–S5), and all missing values were further filled by multiple imputations based on chained equations. To ascertain lifestyle risk factors relative to a country’s wealth, human development, and expenditure on health, we plotted the lifestyle risk score against the country-level indicators (GDP, HDI, IHDI, GDI, GII, CHE, nurses and midwives density, and national age-standardized suicide rate) at the year of survey data collection, and further showed an ordinary least squares regression line across these point estimates, weighting each country equally for visual orientation.

A composite risk score was constructed by a linear combination of the aforementioned factors, where the coefficients for the combination were obtained from a sufficient dimension reduction (SDR) technique based on the distance-covariance matrix [28]. Instead of giving all the factors an equal weight, this method could assign different coefficients to the factors by considering the relationship among the predictors along with the response. A higher score indicated a higher predisposition to risk behaviors. A latent class analysis based on the eight lifestyle factors was further applied to divide participants into different risk clusters [13]. The number of classes was determined according to the conceptual meaning and model fit indices (i.e., Akaike Information Criterion [AIC] and Bayesian Information Criterion [BIC]).

A series of generalized linear mixed models (GLMM) were used to deal with the hierarchical structure data with individuals nested within clustering areas, and estimate the associations between lifestyle risk factors and STBs, adjusting for age, sex, grade, BMI, proxy of SES, loneliness, sleep problem, legislation and religion, according to the proposed directed acyclic graph (DAG) (Additional file 1: Fig. S2). The collinearity of these included variables was tested by the Spearman correlation coefficient as well as the variance inflation factor (VIF) (Additional file 1: Table S6). Subgroup analyses were conducted across strata of world region (East Asia and Pacific, Latin America and Caribbean, Middle East and North Africa, South Asia, and Sub-Saharan Africa), income status (low-income country [LIC], lower-middle-income country [LMC], and upper-middle-income country [UMC]), survey year (2009–2012, 2013–2015, and 2016–2018), age group (≤ 13, 14, 15, and ≥ 16 years), sex (male and female), grade (junior, middle, and senior), and SES (level 1 [lowest], level 2, level 3, level 4, and level 5 [highest]). Results from regression models were presented as odds ratio (OR) and 95% confidence interval (95% CI). A two-sided *p* value of less than 0.10 was considered significant in between-subgroup heterogeneity, whereas a two-sided *p* value of less than 0.05 was considered significant in other estimates. All analyses were conducted by Stata (version 17.0, StataCorp, College Station, TX, USA) and R Statistical Software (version 4.1.2, Foundation for Statistical Computing, Vienna, Austria). Details of the analytic plan were shown in Additional file 1.

## Results

### Study and sample characteristics

**Fig. 1:**
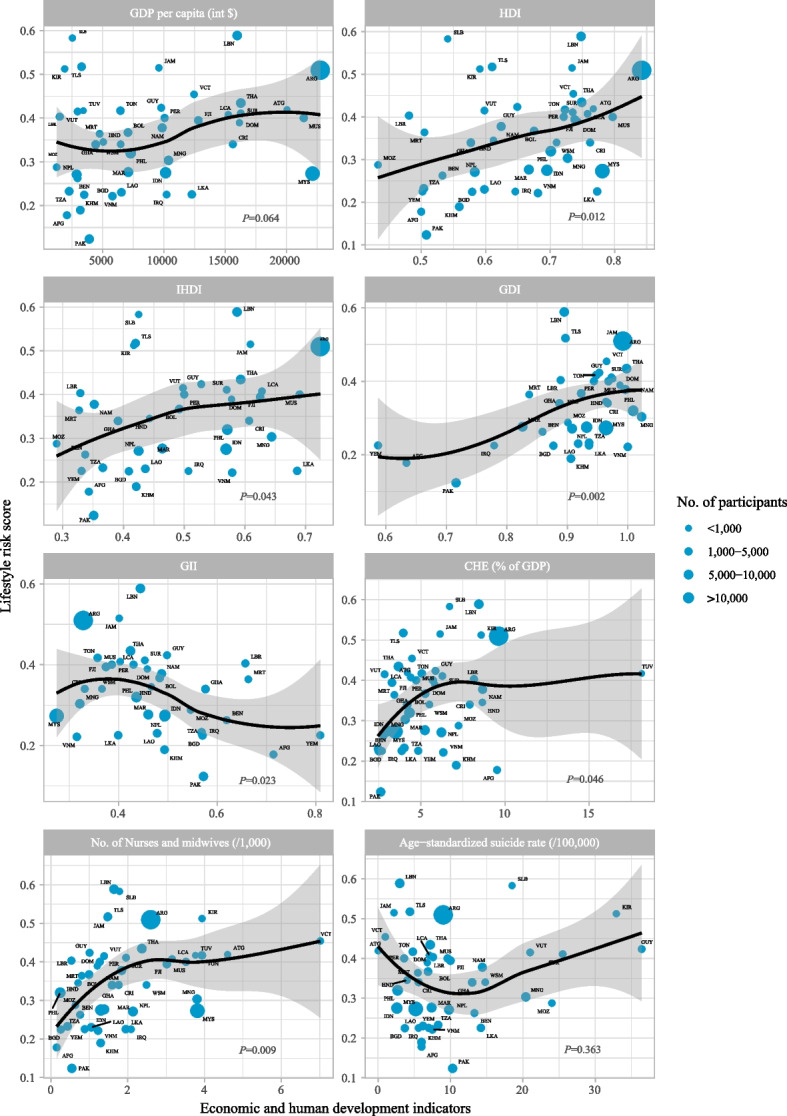
Scatter plots of lifestyle risk scores by economic and human development indicators

Characteristics of the included 45 LMICs are shown in Table 1. The survey-level median participation rate was 79% (IQR 63–89%). The sample size ranged from 943 (Tuvalu) to 56,981 (Argentina). For country-level variables, scatter plots showed that HDI (*P* = 0.012), IHDI (*P* = 0.043), GDI (*P* = 0.002), CHE (*P* = 0.046), and density of nurses and midwives (*P* = 0.009) were positively associated with a lifestyle risk score, whereas GII (*P* = 0.023) was negatively related to the score (Fig. 1).

**Table 1:** Survey characteristics by region and country

Countries	ISO code	Survey year	Participation rate (%)	Sample size	GDP per capita, int $	HDI	IHDI	GDI	GII	CHE, % of GDP	Nurses and midwives per 1,000 people	National age-standardized suicide rate per 100,000 people
**East Asia & Pacific**												
Cambodia	KHM	2013	85	3,806	3,192	0.6	0.4	0.9	0.5	7.1	1.3	6.0
Fiji	FJI	2016	79	3,705	12,821	0.7	0.6^e^	NA	0.4	3.3	3.0^e^	10.0
Indonesia	IDN	2015	94	11,142	10,150	0.7	0.6	0.9	0.5	3.0	1.3	2.6
Kiribati	KIR	2011	85	1,582	1,920	0.6	0.4^c^	NA	NA	8.6	3.9	32.9
Laos	LAO	2015	70	3,683	6,544	0.6	0.4	0.9	0.5	2.5	1.1	6.2
Malaysia	MYS	2012	89	25,507	22,132	0.8	NA	1.0	0.3	3.5	3.8	5.2
Mongolia	MNG	2013	88	5,393	10,384	0.7	0.6	1.0	0.3	4.1	3.8	20.4
Philippines	PHL	2015	79	8,761	7,300	0.7	0.6	1.0	0.4	4.3	0.2	2.7
Samoa	WSM	2017	59	1,955	6,481	0.7	NA	NA	0.4	5.5	2.5^ h^	14.8
Solomon Islands	SLB	2011	85	1,421	2,551	0.5	0.4^c^	NA	NA	6.7	1.8	18.5
Thailand	THA	2015	89	5,894	16,283	0.7	0.6	1.0	0.4	3.7	2.4	7.2
Timor-Leste	TLS	2015	79	3,704	3,298	0.6	0.4	0.9	NA	4.0	1.5	4.4
Tonga	TON	2017	90	3,333	6,467	0.7	NA	0.9	0.4	5.1	3.9^f^	4.8
Tuvalu	TUV	2013	90	943	3,441	NA	NA	NA	NA	18.1	3.8^d^	NA
Vanuatu	VUT	2016	57	2,159	2,973	0.6	0.5	NA	NA	2.8	1.4	21.0
Vietnam	VNM	2013	96	3,331	5,815	0.7	0.6	1.0	0.3	6.3	1.2	7.5
**Latin America & Caribbean**												
Antigua and Barbuda	ATG	2009	67	1,266	20,048	0.8	NA	NA	NA	4.2	4.6^c^	0.0
Argentina	ARG	2018	63	56,981	22,759	0.8	0.7	1.0	0.3	9.6	2.6^ g^	9.0
Bolivia	BOL	2012	88	3,696	7,081	0.7	0.5	0.9	0.5	5.3	1.0^b^	6.9
Costa Rica	CRI	2009	72	2,679	15,631	0.8	0.6	1.0^a^	0.3	7.9	1.8	5.6
Dominican Republic	DOM	2016	63	1,481	16,167	0.7	0.6	1.0	0.5	5.8	1.2	6.8
Guyana	GUY	2010	76	2,392	9,789	0.6	0.5	1.0	0.5	5.9	1.0	36.4
Honduras	HND	2012	79	1,779	5,065	0.6	0.4	1.0	0.5	8.7	0.7^c^	4.0
Jamaica	JAM	2017	60	1,667	9,598	0.7	0.6	1.0	0.4	6.1	1.5	2.2
Peru	PER	2010	85	2,882	10,066	0.7	0.5	0.9	0.4	4.7	1.3	3.6
Saint Lucia	LCA	2018	77	1,970	15,261	0.8	0.6	1.0	0.4	4.4	3.2^ g^	7.1
Saint Vincent and the Grenadines	VCT	2018	78	1,877	12,466	0.7	NA	1.0^i^	NA	4.5	7.0	1.0
Suriname	SUR	2016	83	2,126	16,280	0.7	0.6	1.0	0.5	6.3	2.0	25.5
**Middle East & North Africa**												
Iraq	IRQ	2012	88	2,038	10,234	0.6	0.5^c^	0.8	0.6	2.7	2.1^c^	5.6
Lebanon	LBN	2017	82	5,708	15,988	0.7	0.6^e^	0.9	0.4	8.4	1.6	3.0
Morocco	MAR	2016	91	6,745	7,106	0.7	0.5^e^	0.8	0.5	5.2	1.4^ g^	7.4
Yemen	YEM	2014	75	2,655	NA	0.5	0.3	0.6	0.8	4.8	0.9	7.0
**South Asia**												
Afghanistan	AFG	2014	79	2,579	2,102	0.5	0.3	0.6	0.7	9.5	0.1	6.0
Bangladesh	BGD	2014	91	2,989	3,512	0.6	0.4	0.9	0.6	2.5	0.3	3.7
Nepal	NPL	2015	69	6,529	2,896	0.6	0.4	0.9	0.5	6.2	2.1^d^	9.8
Pakistan	PAK	2009	76	5,192	3,931	0.5	0.4	0.7	0.6	2.6	0.5	10.3
Sri Lanka	LKA	2016	89	3,262	12,287	0.8	0.7	0.9	0.4	3.9	1.9	14.2
**Sub-Saharan Africa**												
Benin	BEN	2016	78	2,536	2,961	0.5	0.3	0.9	0.6	2.8	0.8	13.3
Ghana	GHA	2012	76	3,632	4,443	0.6	0.4	0.9	0.6^b^	4.2	1.6^c^	13.0
Liberia	LBR	2017	71	2,744	1,516	0.5	0.3	0.9	0.7	8.2	0.5^ h^	7.6
Mauritania	MRT	2010	70	2,063	4,767	0.5	0.3	0.8	0.7	3.4	0.8	5.5
Mauritius	MUS	2017	84	3,012	21,415	0.8	0.7	1.0	0.4	5.7	3.5	9.6
Mozambique	MOZ	2015	80	1,918	1,263	0.4	0.3	0.9	0.5	7.2	0.6	24.0
Namibia	NAM	2013	89	4,531	9,872	0.6	0.4	1.0	0.5	8.7	1.8	14.4
United Republic of Tanzania	TZA	2014	87	3,793	2,285	0.5	0.4	0.9	0.6	4.0	0.4	8.3

*CEH* Current Health Expenditure, *GDI* Gender Development Index, *GDP* Gross Domestic Product, *GII* Gender Inequality Index, *HID* Human Development Index, *IHDI* Inequality-adjusted HDI, int international, *ISO* International Organization for Standardization, *NA* Not applicable

^a^Value at 2010

^b^Value at 2011

^c^Value at 2013

^d^Value at 2014

^e^Value at 2015

^f^Value at 2016

^g^Value at 2017

^h^Value at 2018

^i^Value at 2018

Detailed characteristics of the 229,041 adolescents were presented in Table 2. A weighted proportion of 67.57% of the participants were from LMC, and 52.35% of the participants were from East Asia and the Pacific region. The weighted proportions for each age group were relatively balanced (25.93%, 23.54%, 20.56%, and 29.97 for ≤ 13, 14, 15, and ≥ 16 years). 51.47% of the participants were male students and 43.96% were in junior education. A 77.12% weighted proportion of adolescents had two or more co-occurring risk behaviors. We further divided the lifestyle risk score into three groups by tertiles and observed that a high proportion of adolescents with unfavorable lifestyles were from UMC, Latin America, and the Caribbean, with older age, male students, and with lower SES **(**Additional file 1: Table S7). The weighted proportion of adolescents with each single risk behavior was presented in Additional file 1: Table S8.

**Table 2:** Characteristics of the total population in this study

Characteristic	Total	
	**Unweighted *N***	**Weighted %**
***N***	229,041	100
**Country-level factors**		
*** Income status***		
LIC	23,905	13.01
LMC	75,581	67.57
UMC	129,555	19.42
*** World region***		
East Asia & Pacific	86,319	52.35
Latin America & Caribbean	80,796	8.88
Middle East & North Africa	17,146	9.62
South Asia	20,551	17.50
Sub-Saharan Africa	24,229	11.65
*** Survey year***		
2009–2012	56,129	16.82
2013–2015	71,651	69.91
2016–2018	101,261	13.27
**Demographic characteristics**		
*** Age, years***		
≤ 13	50,395	25.93
14	49,356	23.54
15	49,287	20.56
≥ 16	78,708	29.97
Missing	1,295	
*** Sex***		
Male	109,258	51.47
Female	117,260	48.53
Missing	2,523	
*** Body mass index, kg/m***^***2***^		
Level 1 (lowest)	45,915	34.77
Level 2	45,864	27.40
Level 3	45,678	21.73
Level 4 (highest)	45,625	16.10
Missing	45,959	
*** Education level (grade)***		
Junior	92,560	43.96
Middle	81,524	30.69
Senior	51,387	25.35
Missing	3,570	
*** Proxy of SES***		
Highest	121,741	49.27
High	43,491	18.28
Medium	48,458	26.20
Low	7,937	3.60
Lowest	5,300	2.65
Missing	2,114	
**Lifestyle risk factors**		
*** Co-occurring risk behaviours***		
None	4,384	2.68
One-single	31,368	20.20
Two or more	161,030	77.12
Missing	32,259	
*** Fruit consumption***		
Daily	125,510	63.03
Not daily	101,634	36.97
Missing	1,897	
*** Vegetables consumption***		
Daily	15,3147	78.64
Not daily	61,634	21.36
Missing	14,260	
*** Soft drink consumption***		
Not daily	137,380	61.80
Daily	89,984	38.20
Missing	1,677	
*** Fast food consumption***		
≤ 1 day/wk	169,858	73.10
> 1 day/wk	57,740	26.90
Missing	1,443	
*** Tobacco smoking***		
< 1 day/month	189,926	86.86
≥ 1 day/month	38,316	13.14
Missing	799	
*** Alcohol drinking***		
< 1 day/month	164,339	86.42
≥ 1 day/month	54,617	13.58
Missing	10,085	
*** Physical activity for at least 1 h per day***		
Daily	35,461	15.29
Not daily	189,197	84.71
Missing	4,383	
*** Sedentary behaviour***		
≤ 4 h/d	183,406	87.01
> 4 h/d	40,517	12.99
Missing	5,118	
**Suicidality**		
*** Suicidal ideation***		
No	205,389	92.63
Yes	18,220	7.37
Missing	5,432	
*** Suicidal plan***		
No	205,525	94.19
Yes	15,416	5.81
Missing	8,100	
*** Suicidal attempt***		
No	201,968	95.41
Yes	13,747	4.59
Missing	13,326	

*LIC* Low-income country, *LMC* Lower-middle-income country, *UMC* Upper-middle-income country, *SES* Socioeconomic status

The weighted prevalence of suicidal ideation, plan, and attempt was 7.37%, 5.81%, and 4.59%, respectively. The weighted prevalence of each STB by country was presented in Additional file 1: Table S9.

### Lifestyle risk score and STB

The pooled analysis showed that compared with the favorable lifestyle group, intermediate and unfavorable groups had 1.24-fold (95%CI 1.13–1.36) and 1.48-fold (95%CI 1.30–1.69) higher odds of suicidal ideation; 1.22-fold (95%CI 1.12–1.33) and 1.53-fold (95%CI 1.34–1.75) greater odds of suicidal plan; and 1.31-fold (95%CI 1.16–1.49) and 3.11-fold (95%CI 2.64–3.65) increased odds of suicidal attempt, respectively (Table 3). In terms of individual countries, the strongest nexuses for ideation, plan, and attempt were observed in Benin, Antigua and Barbuda, and Tuvalu, respectively (Additional file 1: Fig. S3).

**Table 3:** Association between clustering of risk behaviours and suicidality in the overall samples

	**Suicidal ideation**		**Suicidal plan**		**Suicidal attempt**	
	**OR (95%CI)**	***P***	**OR (95%CI)**	***P***	**OR (95%CI)**	***P***
***Lifestyle risk score***						
Favorable	1.00		1.00		1.00	
Intermediate	1.24 (1.13–1.36)	< 0.001	1.22 (1.12–1.33)	< 0.001	1.31 (1.16–1.49)	< 0.001
Unfavorable	1.48 (1.30–1.69)	< 0.001	1.53 (1.34–1.75)	< 0.001	3.11 (2.64–3.65)	< 0.001
Continuous	1.22 (1.14–1.30)	< 0.001	1.24 (1.16–1.32)	< 0.001	1.82 (1.68–1.98)	< 0.001
***Lifestyle risk cluster***						
H–L cluster	1.00		1.00		1.00	
V-F cluster	1.43 (1.24–1.65)	< 0.001	1.42 (1.19–1.69)	< 0.001	2.43 (1.62–3.64)	< 0.001
D-F cluster	1.19 (0.83–1.70)	0.224	1.25 (0.89–1.74)	0.130	2.85 (1.35–6.03)	0.001
S-A cluster	1.26 (1.15–1.37)	< 0.001	1.20 (1.07–1.34)	0.001	1.18 (1.04–1.34)	0.010

*OR* odds ratio, *CI* Confidence interval, *H–L* Healthy lifestyles, *V-F* Insufficient intake of vegetables and fruit, *D-F* Frequent consumption of soft drink and fast food, *S-A* Tobacco smoking and alcohol drinking

**Fig. 2:**
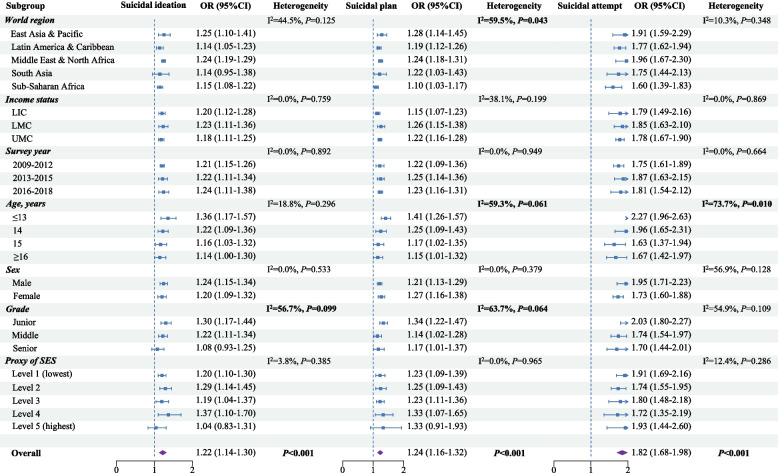
Subgroup analyses on associations of lifestyle risk score with suicidal thoughts and behaviours (LIC Low-income country, LMC Lower-middle-income country, UMC Upper-middle-income country, SES Socioeconomic status, OR Odds ratio, CI Confidence interval)

Subgroup analyses (Fig. 2) showed that the odds of suicidal ideation per additional score level were discrepant among different education levels. The odds were highest in the junior-education group (OR = 1.30, 1.17–1.44) whereas lowest in the senior-education group (OR = 1.08, 95%CI 0.93–1.25). The odds of suicidal plans per additional score level were found heterogeneous in different world regions, age groups, and education levels, with the highest odds in East Asia and Pacific region (OR = 1.28, 95%CI 1.14–1.45), under 13 years of age (OR = 1.41, 95%CI 1.26–1.57), and junior-education group (OR = 1.34, 95%CI 1.22–1.47). The odds of suicidal attempt per additional score level were observed heterogeneous amid different age groups, with the largest odds in the ≤ 13 years of age group (OR = 2.27, 95%CI 1.96–2.63).

### Lifestyle risk clusters and STB

A 4-class solution that provided the most conceptually coherent description of unfavorable lifestyles, was chosen as the most appropriate solution. Class 1 accounted for 46.1% of the adolescents reporting the highest probabilities of healthy lifestyles (H–L cluster), including daily fruit intake (probability (Pr) = 0.893), daily vegetable intake (Pr = 0.907), tobacco smoking of < 1 day/month (Pr = 0.942), alcohol drinking of < 1 day/month (Pr = 0.921), and sedentary behavior of ≤ 4 h/day (Pr = 0.897). Class 2 comprising 32.7% of adolescents, was mainly characterized by insufficient intake of vegetables (Pr = 0.489) and fruit (Pr = 0.819), naming V-F cluster. Class 3 contained one-tenth of adolescents (9.2%) who had frequent consumption of soft drinks (Pr = 0.807) and fast food (Pr = 0.575), calling D-F cluster. Class 4, consisted of 12.0% of adolescents who were most likely to have tobacco smoking (Pr = 0.462) and alcohol drinking behaviors (Pr = 0.919), labelling S-A cluster (Table 4).

**Table 4:** Four-class model: estimated probabilities by latent class membership

lifestyle risk factor	Class 1 (H–L cluster)	Class 2 (V-F cluster)	Class 3 (D-F cluster)	Class 4 (S-A cluster)
**N, %**	105,653 (46.1)	74,791 (32.7)	21,138 (9.2)	27,459 (12.0)
***Fruit intake***				
Daily	**0.893**	0.181	0.767	0.216
Not daily	0.107	**0.819**	0.233	0.784
***Vegetable intake***				
Daily	**0.907**	0.511	0.820	0.442
Not daily	0.093	0.489	0.180	**0.558**
***Soft drink consumption***				
Not daily	0.544	**0.805**	0.193	0.702
Daily	0.457	0.195	**0.807**	0.298
***Fast food consumption***				
≤ 1 day/wk	0.758	0.842	0.425	**0.792**
> 1 day/wk	0.242	0.158	**0.575**	0.208
***Tobacco smoking***				
< 1 day/month	**0.942**	0.919	0.577	0.539
≥ 1 day/month	0.058	0.081	0.423	**0.462**
***Alcohol drinking***				
< 1 day/month	**0.921**	0.900	0.513	0.081
≥ 1 day/month	0.079	0.100	0.487	**0.919**
***Physical activity***				
Daily	0.162	0.133	**0.203**	0.161
Not daily	0.838	**0.867**	0.797	0.839
***Sedentary behaviour***				
≤ 4 h/d	**0.897**	0.825	0.706	0.680
> 4 h/d	0.103	0.175	0.294	**0.320**

Bolded indices are the highest probabilities in the rows

*H–L cluster *Healthy lifestyles*, V-F cluster *Insufficient intake of vegetables and fruit*, D-F cluster *Frequent consumption of soft drink and fast food*, S-A cluster T*obacco smoking and alcohol drinking behaviours

The main analysis showed that compared with H–L cluster, V-F cluster was related to the highest odds of suicidal ideation (OR = 1.43, 95%CI 1.24–1.65) and suicidal plan (OR = 1.42, 95%CI 1.19–1.69). As for suicidal attempts, the highest odd was observed in D-F cluster (OR = 2.85, 95%CI 1.35–6.03) whereas the lowest in S-A cluster (OR = 1.18, 95%CI 1.04–1.34) (Table 3).

**Fig. 3:**
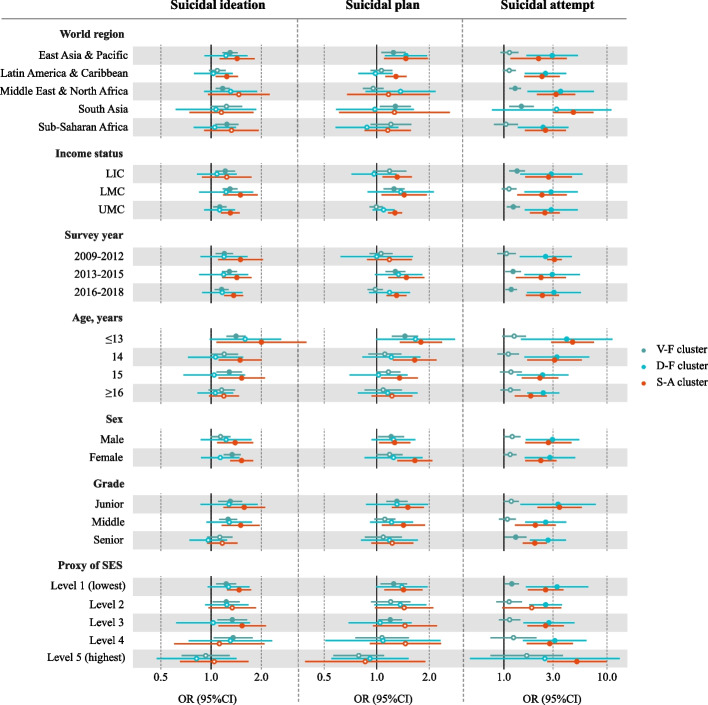
Subgroup analyses on associations of different lifestyle risk clusters with suicidal thoughts and behaviours (LIC Low-income country, LMC Lower-middle-income country, UMC Upper-middle-income country, SES Socioeconomic status, OR Odds ratio, CI Confidence interval, V-F Insufficient intake of vegetables and fruit, D-F Frequent consumption of soft drink and fast food, S-A Tobacco smoking and alcohol drinking)

In subgroup analyses, S-A cluster was associated with the highest odds of suicidal ideation (OR = 2.00, 95%CI 1.08–3.73) and suicidal plan (OR = 1.79, 95%CI 1.35–2.37) in adolescents under 13 years of age, followed by V-F cluster (OR = 1.41, 95%CI 1.24–1.61 for ideation; OR = 1.45, 95%CI 1.22–1.72 for plan). In terms of suicidal attempts, S-A cluster was related to the highest odds among adolescents with the highest SES level (OR = 5.10, 95%CI: 2.62–9.95) (Fig. 3).

Additional analyses of eight single lifestyle factors associated with suicidality were shown in Additional file 1: Table S10. Briefly, we found that tobacco smoking, alcohol drinking, and sedentary behaviors were the first three risk factors for STBs with the strongest and most significant effect sizes.

## Discussion

The present study including a large sample of young adolescents from 45 LMICs provides a cross-national estimate of the positive association between clustering of lifestyle risk factors and STBs. The findings of this study provide insights into the modifiable lifestyle-oriented initiatives on suicide prevention for teenagers.

Unfavorable lifestyle behaviors have been widely reported to co-occur and cluster together among young adolescents. In LMICs, the proportions of co-occurring risk behaviors were varying, with the lowest proportion found in Indian adolescents (19%) [29], whereas the highest proportion in Chinese adolescents (85%) [23]. Any comparison across nations was limited since the estimates were biased by country heterogeneity, risk behaviors component, instruments used, and analytic methods. In this study, we ascertained multiple correlates of unhealthy lifestyles, including income status, sex, age, and education level. Consistent with prior research, unhealthy behaviors were more prevalent in lower socioeconomic groups [30], male adolescents [31], and senior students [32]. The above evidence indicated that interventions targeted at unhealthy lifestyles should be sex-, age-, and socioeconomic-specific.

This study strongly supported that the clustering of lifestyle risk behaviors were associated with an increased risk of adolescent STBs. Several plausible explanations involving biology and psychosociology have been proposed to underline such nexus. For biological pathways, tobacco smoking was relevant to the increased levels of nicotine and the decreased activity of the hippocampal serotonergic system [6]; alcohol drinking was involved in STB via alcohol dependence [33]; insufficient consumption of fruit and vegetables was reportedly associated with lower levels of minerals, vitamins, and antioxidants [34, 35]; frequent consumption of fast food and soft drinks was found related to higher levels of sugar, caffeine, and serum C-reactive protein [36–38]; lacking physical activity might reflect lower levels of brain serotonin [39]; the combination of which could deteriorate the adverse effects of systemic inflammation, oxidative stress, impaired emotional regulation on psychological well-being, which in turn increased the risk of STBs [40–42]. For psychological pathways, previous studies have ascertained the associations between smoking and impulsivity [43], drinking and vulnerability [44], physical activity and body image [45], sedentary behavior and psychological distress [46, 47], and the above psychological factors may act as the mediators [44, 48–50], concurrently explaining the relationship of lifestyle risk behaviors with STBs. For sociological pathways, physical activity enhanced the interactions with the natural environment and increased social cohesion [51], while inertia and sedentary behaviors induced social solitude and poor interpersonal relationships [52], which were confirmed determinants for STBs [53].

We interestingly found that clustering of frequent soft drink and fast-food consumption, rather than other lifestyle risk clusters, was strongest associated with suicidal attempts in LMICs. It is remarkable that LMICs have greater availability of nutrient-poor and energy-dense foods, the resultant burden of adolescent malnutrition may increase the risk of suicidality in these countries [54]. In the country-level analyses, we observed that clustering of smoking and alcohol drinking contributed more to STB in the South Asia region. Since the major tobacco production and export countries are located in South Asia and smokeless tobacco products are widely used in South Asians [55], adolescents from such regions are more likely to be exposed to tobacco environments, which further increases the risk of STBs. On the contrary, STB risk in East Asia and Pacific countries was found attributed more to frequent soft drink and fast-food consumption clusters. A potential explanation is that some common sugar-sweetened beverages and fast-food brands are often counterfeited in these countries, where higher levels of sugar, caffeine, salt, and fat are added in these counterfeits that will further induce STBs via the aforementioned biological mechanisms. Another noticeable finding was that the lifestyle-STB risk association was weaker among adolescents living in Sub-Saharan Africa than in other regions. The ascertainment of any plausible explanation was difficult due to different sociocultural backgrounds and limited research. One possible hypothesis was that several social adversities we undetected here such as violence, wars, and displacement were more common in these regions [56], which may negatively bias the association.

Results of the individual-level analyses indicated that female students with clustering of tobacco smoking and alcohol drinking behaviors had higher odds of STBs compared with their male counterparts. It may be related to the fact that females’ smoking and drinking behaviors are sometimes stigmatized in some cultures which in turn intensifies the STB risk [57, 58]. In addition, we observed a stronger lifestyle-STB relationship among those with younger ages and lower grade levels. This finding is supported by similar evidence in prior research. Aseltine, et al. found that youths aged 13 years and younger with heavy episodic drinking (HED) behavior had 2.6 times more likely to attempt suicide, in contrast to 1.2 times among those with the same HED behavior aged 18 years and older [59]. Peltzer, et al. also found that early substance use (initiation < 12 years vs. ≥ 12 years) including tobacco, smoking, and drug was associated with a 12 to 144% higher absolute risk of suicidal ideation and 63 to 291% increased absolute risk of suicidal attempt [60]. Because early adolescence is a period of rapid development change, where organisms are most sensitive to perturbation [59]. Early adolescence can be a susceptible time window where exposure to unfavorable lifestyles contributes more to STB risk. This informs that healthy lifestyles should be formed at an early age as a potential suicide prevention strategy among adolescents.

The strengths of our study included the use of nationally representative data and the large sample size from 45 low- and middle-income countries. The GSHS was implemented via the same standardized methods, such as sampling strategy, data collection procedure, wording, and coding of the core questionnaire, which largely reduced the bias between countries and made the results more comparable. In addition, the lifestyle risk cluster was constructed using a sufficient dimension reduction technique as well as a latent class analysis. The comprehensive and sophisticated analytics made the results reliable and credible.

However, some limitations should be noted in interpreting our findings. First, all data were self-reported, resulting in the potential reporting and recall bias. Second, the precision of the association estimates may be subject to the single-item measurement for all behaviors and STBs variables. Third, data included in this study were collected over a 10-year period across different geographic locations, and thus any direct comparisons between countries should be treated with caution. Fourth, the timeframe of different lifestyle behaviors in the survey did not overlap completely, but lifestyles are sometimes stable and change little within a year. Furthermore, our sample precluded those who did not attend school, making the results not generalizable to off-school adolescents of the same age. Finally, as with other cross-sectional studies, we could not draw any conclusion on the causal inferences of the lifestyle-STB relationship.

## Conclusions

In conclusion, this large cross-national study indicated that lifestyle risk clusters were informative for suicide risk stratification among school-attending adolescents in LMICs. Our findings emphasize the need to initiate modifiable-lifestyle-oriented suicide prevention strategies, considering region-, sex-, age-, and socioeconomic-specific initiatives.

## Supplementary Information

**Additional file 1: Fig. S1.** Schematic diagram for classifying suicidal thoughts and behaviours according to Nock’s algorithm. **Fig. S2.** Directed acyclic graph (DAG) of lifestyle risk factors linking adolescent suicidality. **Fig. S3.** Associations of lifestyle risk score with suicidal thoughts and behaviors across countries. **Table S1.** Characteristics comparison between included and excluded countries. **Table S2. **Overall percentage of missing values across variables of interest. **Table S3.** Country-specific percentage of missing values across variables of interest. **Table S4.** Examination of missing at random (MAR) pattern hypothesis of variables of interest. **Table S5. **Baseline characteristics between participants with any missing values and those without missing values. **Table S6.** Spearman correlation coefficient matrix and VIF of the independent variables. **Table** **S7. **Population characteristics by lifestyle risk category. **Table S8.** The proportion of each lifestyle risk factor by countries. **Table S9.** The prevalence of suicidality by countries. **Table S10. **Association between specific lifestyle risk factor and suicide risk.

## Data Availability

Data access is subject to approval and can be obtained from the World Health Organization (https://www.who.int/teams/noncommunicable-diseases/surveillance/data).
