# Supplementary material for: Clustering of lifestyle risk factors in relation to suicidal thoughts and behaviors in young adolescents: a cross-national study of 45 low- and middle-income countries
Source: BMC Glob Public Health. 2024 Apr 12;2:24. doi: 10.1186/s44263-024-00055-4 (PMC11622970; doi:10.1186/s44263-024-00055-4)
Supplement: Supplementary file 1 — Additional file 1: Fig. S1. Schematic diagram for classifying suicidal thoughts and behaviours according to Nock’s algorithm. Fig. S2. Directed acyclic graph (DAG) of lifestyle risk factors linking adolescent suicidality. Fig. S3. Associations of lifestyle risk score with suicidal thoughts and behaviors across countries. Table S1. Characteristics comparison between included and excluded countries. Table S2. Overall percentage of missing values across variables of interest. Table S3. Country-specific percentage of missing values across variables of interest. Table S4. Examination of missing at random (MAR) pattern hypothesis of variables of interest. Table S5. Baseline characteristics between participants with any missing values and those without missing values. Table S6. Spearman correlation coefficient matrix and VIF of the independent variables. Table S7. Population characteristics by lifestyle risk category. Table S8. The proportion of each lifestyle risk factor by countries. Table S9. The prevalence of suicidality by countries. Table S10. Association between specific lifestyle risk factor and suicide risk. [file 44263_2024_55_MOESM1_ESM.pdf]

Clustering of Lifestyle Risk Factors in Relation to Suicidal Thoughts and Behaviours in Young Adolescents: A Cross-national Study of 45 Low- and Middle-income Countries

Additional file 1:

Detailed analytic plan.....1

Figure S1. Schematic diagram for classifying suicidal thoughts and behaviours according to Nock's algorithm.....3

Figure S2. Directed acyclic graph (DAG) of lifestyle risk factors linking adolescent suicidality 4

Figure S3. Associations of lifestyle risk score with suicidal thoughts and behaviours across countries 5

Table S1. Characteristics comparison between included and excluded countries.....6

Table S2. Overall percentage of missing values across variables of interest 7

Table S3. Country-specific percentage of missing values across variables of interest.....8

Table S4. Examination of missing at random (MAR) pattern hypothesis of variables of interest 10

Table S5. Baseline characteristics between participants with any missing values and those without missing values 11

Table S6. Spearman correlation coefficient matrix and VIF of the independent variables 12

Table S7. Population characteristics by lifestyle risk category 13

Table S8. The proportion of each lifestyle risk factor by countries.....14

Table S9. The prevalence of suicidality by countries 16

Table S10. Association between specific lifestyle risk factor and suicide risk 18

Detailed analytic plan

1. Framework: examine the cluster effects of lifestyle behaviours on adolescent suicidal thoughts and behaviours (STBs) using the Global School-based Student Health Survey (GSHS) dataset which is a cross-national dataset

2. Outcome variables:

STBs for the present study were evaluated by the following three single-item questions: "During the past 12 months, did you ever seriously consider attempting suicide?"; "During the past 12 months, did you make a plan about how you would attempt suicide?"; "During the past 12 months, how many times did you actually attempt suicide?".

- SI (suicide ideation): participants who answered "yes" to the first question but "no" to the other two questions were considered suicidal ideators.
- SP (suicide plan): participants who answered "yes" to the first two questions but "no" to the third question were considered suicidal planners
- SA (suicide attempt): participants who ever attempted suicide were considered suicidal attempters

3. Predictors: cluster of lifestyle risk
   - Included lifestyle behaviours: fruit consumption (daily vs. not daily), vegetables consumption (daily vs. not daily), soft drink consumption (not daily vs. daily), fast food consumption (⩽1 day/wk vs. >1 day/wk), tobacco smoking (<1 day/month vs. ⩾ 1 day/month), alcohol drinking (< 1 day/month vs. ⩾ 1 day/month), physical activity (daily vs. not daily), and sedentary time (⩽ 4 hours/d vs. > 4 hours/d). The dichotomy of each behaviour was performed according to WHO guidelines or previous studies.
   - sufficient dimension reduction (SDR) technique: lifestyle risk sore
   - latent class analysis (LCA): risk cluster

4. Confounders:

Confounders were selected based on previous literature and a directed acyclic graph (DAG) (Figure S2).

Country-level indicators: Gross Domestic Product (GDP) per capita, the Social Development Index (SDI), the Human Development Index (HDI), Gender Development Index (GDI), total health expenditure (THE) per capita, and national prevalence of crude suicide rate at the survey year, religious background, and legal background.

Individual-level indicators: Age, sex, grade, BMI, proxy of socioeconomic status (SES), loneliness, sleep problem.

5. Missingness
   - Assumption: missing at random (MAR)
   - Imputations: multiple imputations based on chained equations

6. Association analyses between lifestyle risk and suicide
   - Data weighted: all estimates were weighted using the survey's strata, weights, and primary sampling units (PSUs)
   - Baseline description
   - Main analysis: generalized linear mixed models (GLMM)
   - Subgroup analysis: World region, Income status, Survey year, Age group, Sex, Grade of education, Proxy of socioeconomic status (SES)
   - Country-specific analysis: Lifestyle-STB association analysis performed in each of the 45 countries
   - Each lifestyle risk and suicide: eight single lifestyle factors associated with suicidality

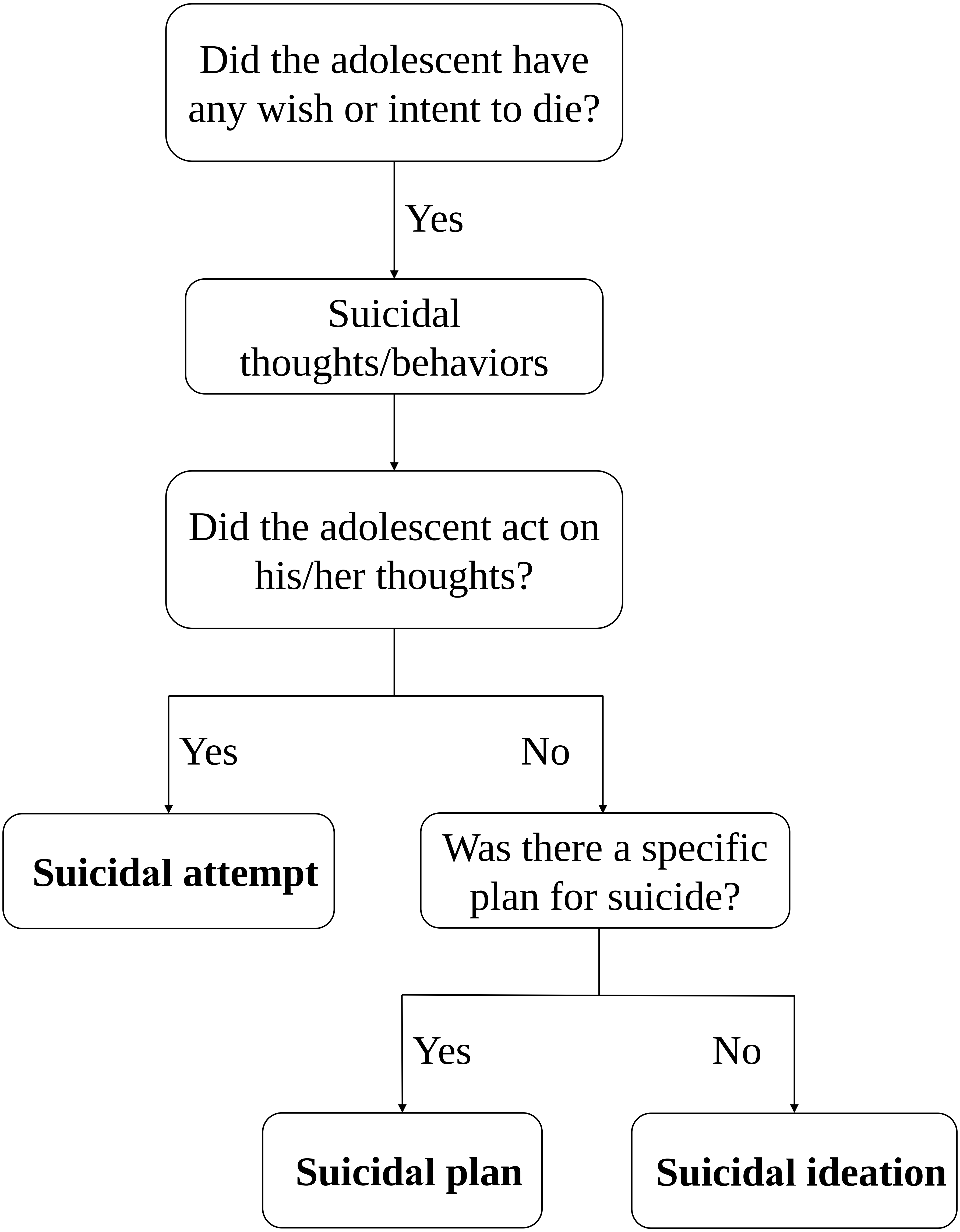

Figure S1. Schematic diagram for classifying suicidal thoughts and behaviours according to Nock's algorithm

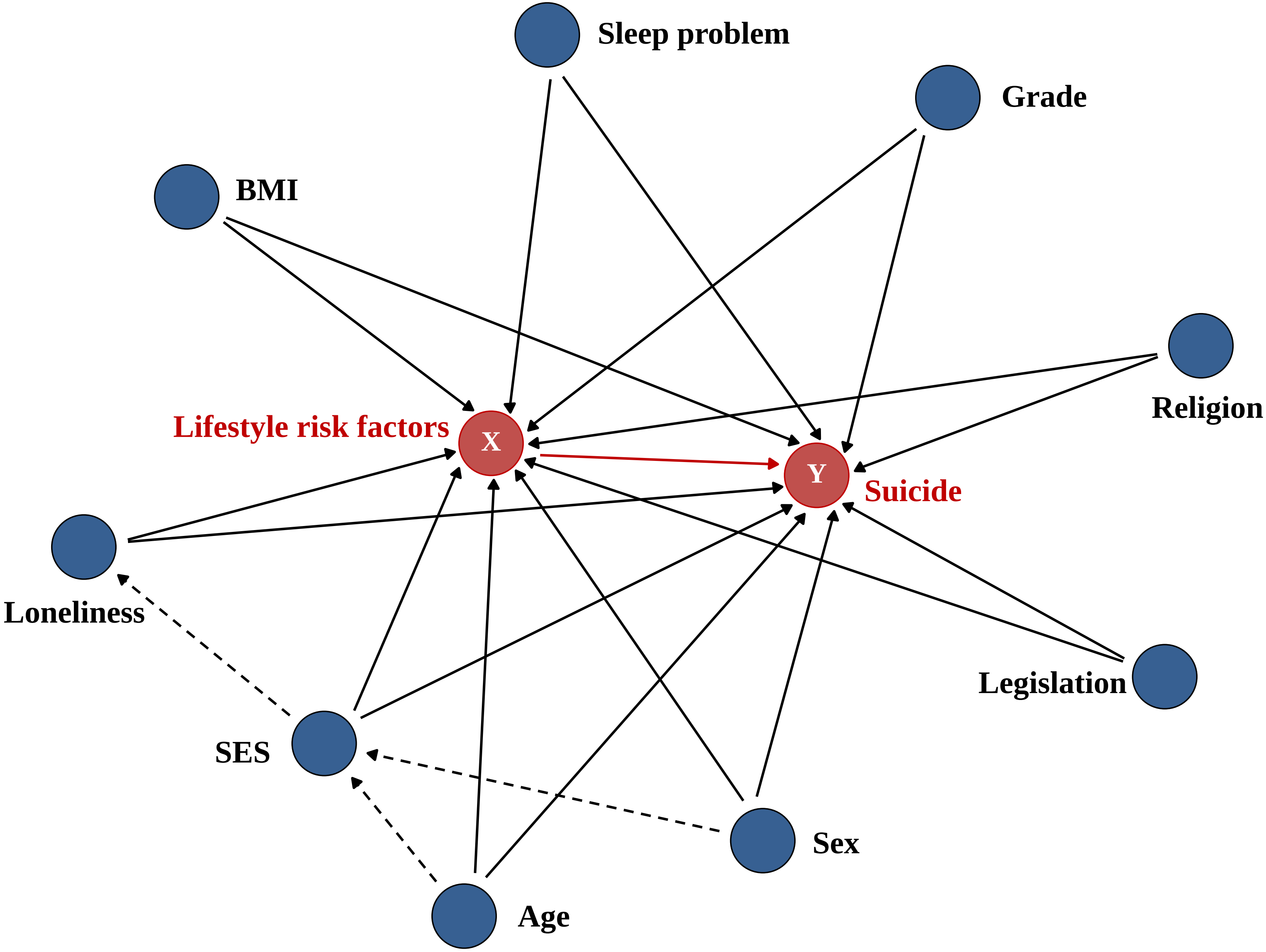

Figure S2. Directed acyclic graph (DAG) of lifestyle risk factors linking adolescent suicidality (BMI, body mass index; SES, socioeconomic status)

	Suicidal ideation	Suicidal plan	Suicidal attempt
**East Asia & Pacific**			
KHM	1.48**	1.27	2.72***
FJI	1.18	1.13	2.64***
IDN	1.41***	1.30***	2.71***
KIR	1.11	1.15	1.69***
LAO	1.55*	1.59**	1.59
MYS	1.27***	1.20**	2.09***
MNG	1.20**	1.09	1.91***
PHL	1.24*	1.46***	1.76***
WSM	0.95	1.05	1.81***
SLB	1.20	1.07	1.65***
THA	1.17	1.28*	2.01***
TLS	1.36**	1.29*	2.94***
TON	1.32*	1.25*	1.70***
TUV	1.58*	1.53*	4.17**
VUT	0.92	1.36**	1.38*
VNM	1.16	1.15	1.68**
**Latin America & Caribbean**			
ATG	1.24	1.91***	2.00***
ARG	1.07	1.19***	1.63***
BOL	1.38**	1.35**	1.89***
CRI	1.50**	1.29	1.60**
DOM	1.19	1.17	1.81**
GUY	1.20	1.19	1.92***
HND	1.25	1.34*	1.94***
JAM	1.12	1.10	1.65***
PER	1.15	1.11	1.92***
LCA	0.97	1.09	1.92***
VCT	1.17	1.17	1.37*
SUR	1.17	1.15	2.83***
**Middle East & North Africa**			
IRQ	1.21	1.26*	1.71***
LBN	1.32**	1.38**	2.28***
MAR	1.26**	1.25**	2.14***
YEM	1.24*	1.28**	2.22***
**South Asia**			
AFG	1.27*	1.08	2.35***
BGD	0.92	1.11	1.48
NPL	1.21*	1.18	2.04***
PAK	1.12	1.46**	1.63***
LKA	1.41***	1.45**	1.86**
**Sub-Saharan Africa**			
BEN	1.61***	1.31*	1.19
GHA	1.09	1.05	1.59***
LBR	1.22*	1.06	1.73***
MRT	1.23*	1.09	1.96***
MUS	1.13	1.13	2.25***
MOZ	1.02	0.99	1.25
NAM	1.38***	1.23**	1.23**
TZA	1.13	1.14	1.96***

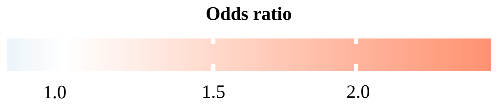

Figure S3. Associations of lifestyle risk score with suicidal thoughts and behaviours across countries (*P<0.05, **P<0.01; ***P<0.001)

Table S1. Characteristics comparison between included and excluded countries

	Included countries (N=45)	Excluded countries (N=12)	*P*
***Income status* (%)**			0.850
LIC	16	17	
LMC	42	50	
UMC	42	33	
***World region* (%)**			0.240
East Asia & Pacific	36	8	
Latin America & Caribbean	27	50	
Middle East & North Africa	9	0	
South Asia	11	17	
Sub-Saharan Africa	18	25	
***Survey year* (%)**			0.960
2009-2012	29	33	
2013-2015	36	33	
2016-2018	36	33	
Sample size (median)	2,898	2,818	0.450
Age, years (mean)	15	14	0.340
***Female* (%)**	52	53	0.450
BMI, kg/m^2 (mean)	21	21	0.910
Lowest grade level (%)	41	45	0.270
Lowest SES level (%)	3.3	2.1	0.075

LIC, low-income country; LMC, lower-middle-income country; UMC, upper-middle-income country; BMI, body mass index; SES, socioeconomic status

Table S2. Overall percentage of missing values across variables of interest (N=229,041)

Variable	No. of Missing (n)	Percent (%)
Age	1,295	0.6
Sex	2,523	1.1
Grade level	3,570	1.6
BMI	45,959	20.0
Proxy of SES	2,114	0.9
Fruit consumption	1,897	0.8
Vegetable consumption	14,260	6.2
Soft drink consumption	1,677	0.7
Fast food consumption	1,443	0.6
Tobacco smoking	799	0.4
Alcohol drinking	10,085	4.4
Physical activity	4,383	1.9
Sedentary behaviour	5,118	2.2
Loneliness	2,652	1.2
Sleep problem	5,223	2.3
Suicidal ideation	5,432	2.4
Suicidal plan	8,100	3.5
Suicidal attempt	13,326	5.8

BMI, body mass index; SES, socioeconomic status

Table S3. Country-specific percentage of missing values across variables of interest (45 included countries)

Countries	Age	Sex	Grade	BMI	SES	Loneliness	Sleep	Fruit	Vegetable	Soft drink	Fast food	Smoking	Drinking	PA	SB	SI	SP	SA
AFG	1.4	3.3	3.1	18.1	2.4	5.3	2.5	2.9	4.5	2.8	2.0	0.6	0.0	2.1	3.6	7.2	7.3	1.5
ATG	0.6	3.3	1.9	84.1	1.4	0.8	1.1	0.7	0.9	0.4	1.3	0.2	4.5	2.5	5.9	1.5	1.0	1.3
ARG	0.2	0.9	1.9	36.7	0.9	0.6	0.7	0.6	21.5	0.7	0.4	0.4	3.8	2.5	2.9	2.4	1.3	1.2
BGD	0.2	0.3	0.6	9.6	2.5	0.4	0.4	1.3	1.3	2.0	1.3	0.3	0.7	2.5	3.6	1.4	0.8	0.6
BEN	0.0	0.8	0.2	3.2	0.4	0.2	0.5	0.4	0.4	0.2	0.4	0.1	10.5	0.7	0.6	0.4	0.4	0.6
BOL	4.1	4.4	1.4	9.1	1.3	0.6	1.8	0.8	1.0	0.5	1.0	0.2	3.8	1.4	2.0	1.9	1.3	1.8
KHM	0.2	0.3	0.5	4.1	0.2	0.8	0.2	0.2	0.4	0.2	0.5	0.1	2.4	0.8	1.1	1.7	0.9	0.5
CRI	0.5	0.4	1.1	3.4	0.8	1.0	0.1	0.6	0.2	0.1	0.3	0.2	1.8	0.6	0.8	1.0	0.6	0.5
DOM	1.2	3.4	2.3	19.0	1.4	2.7	1.2	1.3	2.9	1.3	1.4	0.3	5.5	3.0	3.2	4.5	5.1	1.0
FJI	0.9	2.0	3.0	7.5	1.5	1.2	1.1	2.6	1.0	1.2	0.9	0.5	3.3	3.5	3.5	4.8	4.8	1.2
GHA	0.5	1.1	0.4	3.2	0.3	0.4	0.4	0.5	0.9	0.6	0.2	0.0	7.5	1.1	0.9	1.3	2.7	1.2
GUY	0.5	1.3	1.3	4.8	0.7	0.8	0.8	1.6	0.5	1.0	0.8	0.1	5.7	2.2	1.8	2.6	3.3	0.0
HND	0.9	1.7	0.2	5.6	0.7	2.6	0.6	0.4	1.3	1.0	1.5	0.3	4.3	2.1	2.1	2.5	2.3	0.6
IDN	0.2	0.3	0.6	5.3	0.5	1.0	0.4	1.1	0.5	0.6	0.6	0.6	2.0	2.1	2.0	1.5	1.3	0.3
IRQ	1.0	0.5	0.6	6.5	0.8	1.0	0.8	0.4	0.9	1.5	1.1	0.6	1.0	2.4	4.4	2.6	3.5	1.1
JAM	0.5	0.7	1.0	4.9	0.3	0.7	1.3	0.7	1.9	0.8	0.7	0.3	4.6	3.4	4.1	4.0	1.9	1.9
KIR	1.1	0.4	0.9	9.5	0.8	0.3	0.4	1.0	0.9	0.5	0.4	0.4	4.1	0.3	1.1	3.0	1.1	1.4
LAO	0.5	0.7	0.8	4.0	0.3	0.1	0.4	0.2	0.5	0.3	0.4	0.1	2.6	0.7	0.3	0.5	0.5	0.4
LBN	0.3	0.1	0.5	17.0	0.4	2.0	0.8	0.9	0.7	0.4	0.5	0.8	4.0	5.6	6.7	2.4	2.2	0.8
LBR	3.0	4.0	1.6	98.9	3.8	4.0	3.9	2.8	4.5	3.3	3.2	1.4	9.5	10.7	10.6	7.1	7.3	7.0
MYS	0.1	0.2	0.2	2.9	0.2	0.7	0.2	0.3	0.1	0.2	0.1	0.0	0.9	0.6	0.3	0.9	1.5	0.2
MRT	1.7	1.5	1.8	34.6	1.5	1.7	1.8	1.6	3.2	1.7	1.5	0.6	0.0	2.8	4.2	2.5	3.5	2.1
MUS	0.1	0.5	1.1	2.7	1.3	1.3	2.0	0.6	1.1	0.5	0.2	0.1	3.6	1.7	2.0	1.7	1.7	1.5
MNG	0.2	0.4	1.3	2.3	0.5	0.2	0.5	0.5	0.8	0.3	0.8	0.0	1.6	0.9	0.5	1.2	0.6	0.8
MAR	1.7	2.6	3.0	10.3	3.1	2.8	1.3	1.9	2.1	1.4	0.9	1.1	39.6	2.2	4.2	4.7	5.4	1.8
MOZ	1.5	2.8	1.5	75.9	2.6	1.9	2.5	2.5	1.8	1.3	1.7	0.8	7.5	3.1	2.5	4.8	4.9	3.0
NAM	1.0	1.4	2.3	6.3	1.8	1.1	1.0	1.1	1.2	1.1	0.2	0.2	5.5	1.1	1.5	3.2	1.8	1.6

NPL	0.7	1.6	1.4	11.3	0.8	2.2	0.6	0.8	1.2	0.8	1.0	0.3	2.2	1.7	1.6	2.1	1.9	0.5
PAK	0.1	0.2	0.1	3.9	1.1	1.1	0.5	0.4	0.3	0.6	0.3	0.0	0.1	1.2	0.9	1.5	1.0	0.0
PER	0.5	0.6	0.7	9.1	0.0	0.5	0.6	0.0	0.2	0.1	0.1	0.0	5.2	0.5	0.2	0.8	0.6	1.0
PHL	0.1	0.0	1.2	14.9	0.1	2.2	0.1	0.7	0.1	0.3	0.1	0.0	1.7	1.2	1.1	2.2	2.2	0.2
LCA	0.3	0.9	1.2	94.2	0.3	0.8	2.3	0.8	2.3	0.9	1.6	0.3	6.2	2.1	2.5	2.1	3.0	2.2
VCT	0.6	0.9	1.5	9.7	1.2	1.1	2.7	0.5	14.6	0.8	0.5	0.1	4.4	2.0	2.3	2.0	2.1	2.2
WSM	1.6	2.6	1.6	9.8	1.5	0.9	1.3	0.5	0.9	1.2	0.5	1.4	4.9	5.1	7.1	5.6	3.8	2.1
SLB	3.8	4.6	5.8	20.1	2.0	1.6	2.8	0.9	2.3	1.3	2.2	0.1	8.2	2.0	2.2	2.6	2.8	3.1
LKA	0.0	0.6	0.4	49.8	0.5	0.4	1.1	0.5	1.1	0.5	0.3	0.1	2.2	0.6	0.7	1.3	1.6	1.3
SUR	0.3	0.7	5.4	2.9	1.0	1.1	0.5	0.3	0.4	1.2	0.6	0.2	7.8	1.4	1.1	2.7	2.3	1.1
THA	0.3	0.5	0.8	5.4	0.2	1.3	1.7	0.9	2.2	0.4	1.0	0.6	3.2	1.0	1.9	4.2	3.8	1.5
TLS	2.0	5.5	2.9	18.4	1.6	3.0	0.9	2.9	1.4	2.1	2.0	0.6	9.4	1.3	1.2	4.2	3.9	0.8
TON	0.2	0.6	0.4	6.3	0.9	0.6	1.3	1.0	0.5	0.5	0.5	0.1	3.7	1.2	1.6	2.7	3.1	2.0
TUV	3.4	0.7	45.0	37.5	1.8	2.6	0.7	2.9	1.5	0.7	1.6	0.5	5.8	3.2	4.7	3.0	2.8	1.7
TZA	0.7	2.0	1.3	92.2	1.4	1.3	1.7	0.9	0.7	0.6	0.6	0.3	4.1	0.8	3.0	3.1	3.0	2.1
VUT	1.1	1.4	4.8	21.5	1.1	2.4	0.6	1.1	1.6	1.3	0.9	0.4	3.5	1.3	1.7	2.9	3.7	1.1
VNM	0.2	0.3	0.5	3.6	0.5	1.6	100	0.4	0.7	0.4	0.4	0.2	4.0	0.3	0.4	1.3	0.0	0.0
YEM	2.3	4.9	3.0	16.0	3.2	2.8	1.5	1.9	3.7	1.8	2.8	0.4	0.0	1.5	2.0	5.0	5.2	1.7

BMI, body mass index; SES, socioeconomic status; PA, physical activity; SB, sedentary behaviour; SI, suicidal ideation; SP, suicidal plan; SA, suicidal attempt

Table S4. Examination of missing at random (MAR) pattern hypothesis of variables of interest (*P*-value)

	Age	Sex	Grade	BMI	SES	Loneliness	Sleep	Fruit	Vegetable	Soft drink	Fast food	Smoking	Drinking	PA	SB	SI	SP	SA
**Age**	NA	0.002	<0.001	1.000	<0.001	0.065	<0.001	0.583	0.130	<0.001	<0.001	<0.001	0.017	<0.001	<0.001	<0.001	<0.001	<0.001
**Sex**	<0.001	NA	0.002	1.000	<0.001	0.001	<0.001	<0.001	0.214	<0.001	<0.001	<0.001	<0.001	<0.001	<0.001	<0.001	<0.001	<0.001
**Grade**	0.021	0.001	NA	<0.001	<0.001	0.327	0.022	0.067	<0.001	<0.001	<0.001	<0.001	<0.001	<0.001	0.024	<0.001	<0.001	<0.001
**BMI**	<0.001	<0.001	<0.001	NA	<0.001	0.161	<0.001	<0.001	<0.001	<0.001	<0.001	<0.001	<0.001	<0.001	<0.001	<0.001	<0.001	<0.001
**SES**	<0.001	0.041	<0.001	0.011	NA	0.024	0.003	0.677	0.944	<0.001	<0.001	<0.001	<0.001	0.002	0.015	<0.001	<0.001	<0.001
**Loneliness**	<0.001	<0.001	<0.001	<0.001	<0.001	NA	<0.001	<0.001	0.118	<0.001	<0.001	<0.001	0.090	<0.001	0.345	<0.001	<0.001	<0.001
**Sleep**	<0.001	0.196	<0.001	<0.001	0.002	<0.001	NA	<0.001	<0.001	0.001	0.001	<0.001	<0.001	<0.001	0.001	<0.001	<0.001	<0.001
**Fruit**	<0.001	0.001	<0.001	0.279	<0.001	0.839	0.032	NA	0.001	<0.001	<0.001	<0.001	0.091	<0.001	<0.001	<0.001	<0.001	<0.001
**Vegetable**	<0.001	<0.001	0.294	<0.001	<0.001	<0.001	<0.001	<0.001	NA	<0.001	<0.001	<0.001	<0.001	<0.001	<0.001	<0.001	<0.001	<0.001
**Soft drink**	0.004	0.002	<0.001	0.096	<0.001	0.490	0.020	0.034	0.223	NA	<0.001	<0.001	0.802	0.001	0.001	<0.001	<0.001	<0.001
**Fast food**	<0.001	0.755	<0.001	0.148	<0.001	0.384	0.491	<0.001	0.543	<0.001	NA	<0.001	0.001	<0.001	<0.001	<0.001	<0.001	<0.001
**Smoking**	<0.001	0.006	<0.001	0.122	0.051	0.097	0.265	0.326	0.174	<0.001	<0.001	NA	0.757	0.037	0.997	0.002	<0.001	<0.001
**Drinking**	<0.001	<0.001	<0.001	0.085	<0.001	<0.001	<0.001	<0.001	0.581	<0.001	<0.001	<0.001	NA	<0.001	0.274	<0.001	<0.001	<0.001
**PA**	0.002	<0.001	<0.001	<0.001	0.041	0.474	0.047	0.392	<0.001	<0.001	<0.001	<0.001	<0.001	NA	<0.001	<0.001	<0.001	<0.001
**SB**	<0.001	<0.001	<0.001	<0.001	<0.001	0.128	<0.001	0.406	<0.001	<0.001	<0.001	<0.001	<0.001	0.074	NA	<0.001	<0.001	<0.001
**SI**	<0.001	<0.001	<0.001	0.487	<0.001	<0.001	<0.001	<0.001	0.004	<0.001	<0.001	<0.001	<0.001	<0.001	<0.001	NA	<0.001	<0.001
**SP**	<0.001	<0.001	0.457	<0.001	<0.001	<0.001	<0.001	<0.001	<0.001	<0.001	<0.001	<0.001	<0.001	<0.001	<0.001	<0.001	NA	<0.001
**SA**	<0.001	<0.001	<0.001	<0.001	<0.001	<0.001	0.004	<0.001	<0.001	<0.001	<0.001	0.095	<0.001	<0.001	<0.001	0.773	<0.001	NA

BMI, body mass index; SES, socioeconomic status; PA, physical activity; SB, sedentary behaviour; SI, suicidal ideation; SP, suicidal plan; SA, suicidal attempt

Table S5. Baseline characteristics between participants with any missing values and those without missing values (%)

	Without missing values (n=143,021)	With any missing values (n=86,020)
**Age, years**		
≤13	21.8	22.6
14	20.9	23.0
15	21.7	21.6
≥16	35.6	32.8
**Gender**		
Male	46.2	51.8
Female	53.8	48.2
**Body mass index, kg/m^2**		
Level 1 (lowest)	25.8	26.3
Level 2	24.7	24.7
Level 3	24.2	24.8
Level 4 (highest)	25.4	24.2
**Education level (grade)**		
Junior	40.7	41.6
Middle	35.7	36.9
Senior	23.5	21.5
**Proxy of SES**		
Highest	51.4	57.5
High	19.9	17.8
Medium	23.1	18.3
Low	3.3	3.8
Lowest	2.2	2.6

BMI, body mass index; SES, socioeconomic status

Table S6. Spearman correlation coefficient matrix and VIF of the independent variables

	Correlation coefficient matrix										VIF
	Lifestyle score	Age	Sex	Grade	BMI	Proxy of SES	Loneliness	Sleep problem	Legislation	Religion	
**Lifestyle score**	1.00										1.08
**Age**	0.13	1.00									1.24
**Sex**	-0.04	-0.02	1.00								1.03
**Grade**	0.08	0.67	0.02	1.00							1.36
**BMI**	0.08	0.00	0.00	0.03	1.00						1.01
**Proxy of SES**	0.01	0.03	-0.01	-0.01	-0.04	1.00					1.02
**Loneliness**	0.12	0.11	0.14	0.09	0.01	0.18	1.00				1.09
**Sleep problem**	0.15	0.13	0.12	0.10	0.02	0.16	0.41	1.00			1.09
**Legislation**	-0.02	-0.05	0.02	0.01	0.01	0.03	0.02	0.01	1.00		1.00
**Religion**	-0.18	-0.05	0.00	-0.03	-0.18	0.02	-0.06	-0.08	0.04	1.00	1.03
**Mean VIF**											1.09

BMI, body mass index; SES, socioeconomic status; VIF, variance inflation factor

Table S7. Population characteristics by lifestyle risk category (weighted %)

**Characteristics**	**Lifestyle risk category**			***P* trend**
	**Favorable**	**Intermediate**	**Unfavorable**	
**Income status**				<0.001
LIC	43.77 (40.82-46.72)	35.75 (32.92-38.58)	20.48 (18.90-22.06)	
LMC	45.28 (43.54-47.02)	33.77 (32.48-35.05)	20.95 (19.85-22.05)	
UMC	28.66 (27.36-29.96)	30.91 (30.06-31.76)	40.43 (39.15-41.71)	
**World region**				
East Asia & Pacific	37.83 (36.15-39.52)	37.71 (36.41-39.01)	24.46 (23.27-25.65)	
Latin America & Caribbean	22.56 (21.14-23.98)	35.13 (34.22-36.05)	42.31 (40.85-43.76)	
Middle East & North Africa	62.02 (58.52-65.53)	14.65 (12.90-16.41)	23.32 (21.08-25.57)	
South Asia	53.40 (50.01-56.80)	30.00 (27.23-32.78)	16.59 (14.30-18.89)	
Sub-Saharan Africa	40.64 (37.99-43.28)	33.89 (31.57-36.21)	25.48 (23.63-27.32)	
**Survey year**				<0.001
2009-2012	51.40 (48.77-54.04)	25.74 (24.19-27.29)	22.86 (21.39-24.33)	
2013-2015	40.68 (39.40-41.96)	36.17 (35.18-37.16)	23.15 (22.04-24.26)	
2016-2018	35.94 (33.48-38.40)	29.05 (27.79-30.30)	35.01 (33.10-36.92)	
**Age, years**				<0.001
≤13	46.93 (45.12-48.74)	33.38 (31.96-34.80)	19.69 (18.34-21.04)	
14	45.31 (43.62-47.01)	33.28 (31.95-34.61)	21.41 (20.17-22.64)	
15	42.78 (41.08-44.49)	32.14 (30.68-33.60)	25.08 (23.42-26.74)	
≥16	34.13 (32.79-35.47)	34.61 (33.32-35.90)	31.26 (29.95-32.57)	
**Gender**				
Male	39.81 (38.58-41.04)	30.67 (29.56-31.77)	29.53 (28.25-30.80)	
Female	44.03 (42.29-45.77)	36.44 (35.25-37.63)	19.53 (18.54-20.52)	
**Body mass index, kg/m^2**				<0.001
Level 1 (lowest)	43.49 (41.96-45.02)	34.11 (32.99-35.23)	22.40 (21.31-23.49)	
Level 2	42.17 (40.70-43.63)	33.34 (32.16-34.51)	24.50 (23.42-25.57)	
Level 3	40.58 (39.36-41.79)	33.16 (31.94-34.39)	26.26 (24.88-27.64)	
Level 4 (highest)	39.78 (38.06-41.50)	32.80 (31.32-34.28)	27.42 (26.06-28.77)	
**Education level (grade)**				<0.001
Junior	44.51 (43.06-45.96)	33.65 (32.53-34.77)	21.84 (20.73-22.96)	
Middle	41.02 (39.38-42.66)	33.30 (31.96-34.64)	25.68 (24.37-26.99)	
Senior	38.28 (36.32-40.23)	33.35 (31.57-35.14)	28.37 (26.63-30.11)	
**Proxy of SES**				<0.001
Highest	45.51 (44.24-46.77)	31.68 (30.72-32.63)	22.82 (21.77-23.86)	
High	36.67 (34.96-38.38)	35.77 (34.16-37.37)	27.56 (26.09-29.03)	
Medium	39.14 (37.07-41.22)	35.92 (34.34-37.50)	24.94 (23.68-26.19)	
Low	37.55 (34.51-40.59)	32.70 (29.44-35.96)	29.75 (27.10-32.41)	
Lowest	42.37 (38.44-46.30)	27.79 (24.10-31.47)	29.84 (27.00-32.68)	

LIC, low-income country; LMC, low- and middle-income country; HIC, high-income country; SES, socioeconomic status

Table S8. The proportion of each lifestyle risk factor by countries (weighted %)

ISO code	Fruit consumption (not daily)	Vegetables consumption (not daily)	Soft drink consumption (daily)	Fast food consumption (>1 day/wk)	Tobacco smoking (≥ 1 day/month)	Alcohol drinking (≥ 1 day/month)	Physical activity (not daily)	Sedentary behaviour (> 4 hours/d)
AFG*	42.27	35.55	37.55	36.79	12.66	NA	90.16	10.03
ATG	32.32	25.77	58.10	30.27	13.66	43.51	77.40	33.17
ARG	69.94	48.58	33.25	19.12	21.26	53.51	83.43	29.45
BGD	50.03	25.43	47.09	36.96	10.61	1.91	59.02	6.89
BEN	36.31	35.77	34.86	18.83	8.31	42.15	70.48	11.32
BOL	29.69	19.46	62.78	29.25	18.76	19.26	86.38	9.69
KHM	47.40	23.42	42.06	6.78	4.16	10.26	92.44	5.75
CRI	39.88	25.35	51.61	26.39	13.20	26.69	81.58	19.05
DOM	40.17	35.73	72.28	23.28	13.38	45.92	87.91	23.68
FJI	35.87	14.82	62.21	34.36	18.86	20.78	79.38	15.03
GHA	37.99	28.02	45.77	41.32	16.59	15.65	87.47	9.47
GUY	25.12	17.21	70.69	31.50	17.36	40.49	84.70	19.11
HND	32.73	30.72	73.57	25.49	14.73	16.33	84.25	12.30
IDN	36.09	17.87	28.02	25.46	13.43	4.74	87.73	10.57
IRQ*	29.08	22.80	53.82	32.66	17.16	NA	85.28	9.87
JAM	40.06	32.59	68.28	34.39	21.06	47.90	76.52	36.92
KIR	45.79	47.36	22.27	22.35	33.58	32.15	82.25	5.87
LAO	37.51	21.70	50.20	16.66	6.88	29.19	83.31	7.39
LBN	24.49	25.62	47.58	46.68	35.24	18.11	85.41	21.72
LBR	38.97	31.39	45.11	23.72	21.85	25.91	87.23	11.81
MYS	32.11	19.38	29.38	14.69	13.37	9.09	85.83	20.54
MRT*	43.53	34.02	50.38	39.61	26.53	NA	87.60	19.84
MUS	39.54	13.14	41.01	26.10	21.35	26.73	81.13	19.82
MNG	59.77	24.42	33.63	36.48	12.58	9.26	74.92	19.45
MAR*	27.32	16.91	32.74	40.70	14.94	NA	89.23	13.70
MOZ	32.53	32.13	56.81	35.06	9.24	13.16	85.55	17.53
NAM	44.28	41.15	46.62	27.73	13.43	33.25	85.65	19.88
NPL	52.32	37.63	33.36	41.44	10.53	6.09	84.93	4.32
PAK*	32.54	9.82	36.28	6.96	10.82	NA	88.48	2.37
PER	27.56	24.47	54.03	23.96	20.55	29.71	84.77	10.17
PHL	37.28	21.27	37.26	18.84	16.48	21.43	92.47	14.03
LCA	31.76	37.99	53.68	39.02	11.82	45.63	78.86	34.69
VCT	78.83	66.87	41.44	41.19	13.52	47.28	81.53	32.68
WSM	25.79	23.02	63.37	41.46	16.74	13.94	78.44	16.16
SLB	33.21	20.80	44.73	41.85	35.28	23.60	83.59	13.43
LKA	34.44	10.14	26.48	20.80	9.49	4.06	84.49	14.38
SUR	27.91	12.39	78.44	34.35	16.17	39.28	81.17	20.33
THA	27.03	14.99	56.17	66.55	15.41	22.52	88.26	30.13
TLS	53.77	41.02	42.12	26.02	32.14	18.43	90.10	7.25
TON	28.75	24.20	59.75	42.99	23.49	15.29	82.06	12.57
TUV	39.57	36.24	51.42	25.69	23.31	17.29	87.84	8.89
TZA	31.52	24.29	45.27	22.76	10.14	5.26	80.02	9.40

VUT	23.54	21.78	38.72	23.82	28.02	19.09	87.29	11.90
VNM	28.06	10.70	30.20	16.45	5.62	24.73	86.14	13.59
YEM*	40.94	29.27	37.20	20.35	18.76	NA	87.66	8.65

* Under-age drinking was forbidden in AFG, IRQ, MRT, MAR, PAK, and YEM.

Table S9. The prevalence of suicidality by countries (weighted %)

Countries	ISO code	Suicidal ideation	Suicidal plan	Suicidal attempt
Afghanistan	AFG	11.73 (9.10-15.00)	10.02 (8.16-12.25)	6.92 (5.00-9.51)
Antigua and Barbuda	ATG	9.17 (7.46-11.22)	9.91 (8.07-12.12)	6.49 (5.08-8.27)
Argentina	ARG	10.51 (9.90-11.14)	7.72 (7.28-8.19)	8.85 (8.30-9.42)
Bangladesh	BGD	3.04 (2.19-4.23)	4.20 (2.87-6.12)	1.97 (1.22-3.15)
Benin	BEN	6.12 (4.71-7.91)	6.85 (5.73-8.17)	6.31 (5.01-7.92)
Bolivia	BOL	5.82 (4.94-6.86)	5.51 (4.51-6.72)	10.13 (8.99-11.40)
Cambodia	KHM	3.32 (2.79-3.95)	5.39 (4.42-6.55)	2.86 (2.41-3.39)
Costa Rica	CRI	5.93 (5.00-7.03)	3.19 (2.69-3.77)	3.67 (3.09-4.35)
Dominican Republic	DOM	8.48 (6.24-11.44)	6.12 (4.34-8.57)	9.18 (6.53-12.76)
Fiji	FJI	7.44 (6.44-8.58)	9.02 (7.67-10.59)	5.66 (4.19-7.62)
Ghana	GHA	6.88 (5.69-8.29)	8.93 (7.39-10.75)	9.32 (7.55-11.45)
Guyana	GUY	23.51 (21.26-25.93)	23.40 (21.06-25.91)	NA
Honduras	HND	7.27 (6.15-8.56)	7.34 (5.92-9.05)	9.97 (8.31-11.92)
Indonesia	IDN	3.73 (3.20-4.34)	3.91 (3.50-4.36)	1.45 (1.14-1.85)
Iraq	IRQ	7.82 (6.54-9.32)	7.29 (6.11-8.69)	8.26 (7.22-9.44)
Jamaica	JAM	12.41 (10.57-14.53)	12.49 (10.24-15.16)	11.57 (9.69-13.76)
Kiribati	KIR	11.18 (9.71-12.84)	12.21 (10.79-13.78)	20.96 (18.13-24.12)
Lao People's Democratic Republic	LAO	1.83 (1.44-2.33)	2.81 (2.32-3.40)	0.78 (0.48-1.26)
Lebanon	LBN	7.18 (6.45-7.98)	3.99 (3.52-4.51)	4.67 (3.92-5.56)
Liberia	LBR	11.03 (9.26-13.09)	16.50 (14.89-18.25)	13.48 (10.77-16.76)
Malaysia	MYS	4.78 (4.36-5.24)	3.26 (2.93-3.62)	2.50 (2.22-2.82)
Mauritania	MRT	8.39 (6.20-11.27)	8.15 (6.13-10.75)	6.24 (4.39-8.81)
Mauritius	MUS	7.53 (6.27-9.02)	7.50 (6.44-8.73)	7.08 (6.02-8.31)
Mongolia	MNG	15.10 (13.73-16.58)	7.65 (6.85-8.53)	6.61 (5.84-7.47)
Morocco	MAR	7.90 (7.14-8.73)	7.71 (6.76-8.78)	6.22 (5.41-7.13)
Mozambique	MOZ	11.70 (8.36-16.15)	11.99 (8.45-16.74)	6.54 (5.50-7.76)
Namibia	NAM	7.64 (6.76-8.63)	8.94 (8.03-9.95)	9.64 (8.31-11.14)
Nepal	NPL	8.82 (7.56-10.26)	9.48 (8.11-11.06)	3.91 (2.93-5.20)
Pakistan	PAK	7.23 (6.23-8.38)	7.53 (6.49-8.73)	NA
Peru	PER	7.57 (6.53-8.78)	4.61 (3.74-5.67)	9.46 (8.28-10.78)
Philippines	PHL	3.86 (3.24-4.60)	4.01 (3.48-4.62)	5.40 (4.78-6.10)
Saint Lucia	LCA	13.25 (11.67-14.99)	9.55 (8.39-10.86)	9.74 (8.03-11.77)
Saint Vincent and the Grenadines	VCT	14.47 (13.03-16.05)	13.19 (11.24-15.41)	10.37 (8.68-12.34)
Samoa	WSM	8.87 (7.20-10.87)	9.11 (7.68-10.78)	13.61 (11.65-15.83)
Solomon Islands	SLB	9.55 (8.22-11.07)	8.65 (6.69-11.12)	11.11 (8.84-13.88)
Sri Lanka	LKA	6.56 (5.46-7.87)	3.65 (2.96-4.49)	2.26 (1.78-2.88)
Suriname	SUR	9.08 (7.96-10.35)	7.01 (6.14-8.00)	7.14 (5.62-9.03)
Thailand	THA	6.37 (5.52-7.33)	6.88 (5.86-8.06)	6.58 (4.79-8.98)
Timor-Leste	TLS	6.02 (5.21-6.94)	5.80 (4.90-6.85)	3.57 (2.84-4.47)
Tonga	TON	5.45 (4.67-6.34)	7.85 (6.19-9.91)	5.24 (4.36-6.28)
Tuvalu	TUV	5.33 (5.33-5.33)	8.04 (8.04-8.04)	2.90 (2.90-2.90)
United Republic of Tanzania	TZA	9.91 (8.40-11.65)	6.32 (5.05-7.87)	3.18 (2.49-4.06)
Vanuatu	VUT	7.37 (6.07-8.93)	9.59 (8.23-11.15)	6.02 (4.99-7.25)
Vietnam	VNM	16.90 (14.88-19.13)	NA	NA

Yemen	YEM	10.00 (9.01-11.08)	9.35 (7.57-11.50)	5.54 (4.43-6.92)

Table S10. Association between specific lifestyle risk factor and suicide risk

Lifestyle risk factor	Suicidal ideation		Suicidal plan		Suicidal attempt	
	**OR (95%CI)**	***P***	**OR (95%CI)**	***P***	**OR (95%CI)**	***P***
Fruit intake						
Daily	1.00		1.00		1.00	
Not daily	1.27 (1.18-1.37)	**<0.001**	1.22 (1.10-1.35)	**<0.001**	1.10 (1.03-1.19)	**0.008**
Vegetable intake						
Daily	1.00		1.00		1.00	
Not daily	1.17 (1.09-1.27)	**<0.001**	1.20 (1.08-1.33)	**0.001**	1.20 (1.11-1.31)	**<0.001**
Soft drink consumption						
Not daily	1.00		1.00		1.00	
Daily	1.00 (0.94-1.07)	0.999	1.01 (0.92-1.12)	0.761	1.29 (1.20-1.39)	**<0.001**
Fast food consumption						
≤ 1 day/wk	1.00		1.00		1.00	
>1 day/wk	1.00 (0.90-1.11)	0.971	1.07 (0.99-1.16)	0.090	1.34 (1.16-1.54)	**<0.001**
Tobacco smoking						
< 1 day/month	1.00		1.00		1.00	
≥ 1 day/month	1.39 (1.24-1.56)	**<0.001**	1.41 (1.21-1.65)	**<0.001**	3.33 (2.76-4.01)	**<0.001**
Alcohol drinking						
<1 day/month	1.00		1.00		1.00	
≥ 1 day/month	1.24 (1.12-1.37)	**<0.001**	1.22 (1.08-1.39)	**0.001**	2.37 (1.91-2.93)	**<0.001**
Physical activity						
Daily	1.00		1.00		1.00	
Not daily	1.14 (1.03-1.26)	**0.009**	1.11 (0.99-1.25)	0.069	1.02 (0.84-1.24)	0.850
Sedentary behaviour						
≤ 4 hours/d	1.00		1.00		1.00	
> 4 hours/d	1.28 (1.13-1.44)	**<0.001**	1.21 (1.09-1.33)	**<0.001**	1.57 (1.35-1.83)	**<0.001**

OR, odds ratio; CI, confidence interval
